# Hamstring Tendon Grafts for Anterior Cruciate Ligament Reconstruction: The Effect of a 180° Twist Angle on Tensile Properties

**DOI:** 10.3390/bioengineering12101105

**Published:** 2025-10-14

**Authors:** Jure Serdar, Ana Pilipović, Giuseppe Filardo, Slavica Martinović, Anita Galić Mihić, Mihovil Plečko, Ozgur Basal, Tomislav Smoljanović

**Affiliations:** 1 Department of Orthopaedic Surgery, University Hospital Centre Zagreb, 10000 Zagreb, Croatia; mihovil.plecko@kbc-zagreb.hr (M.P.); tomislav.smoljanovic@kbc-zagreb.hr (T.S.); 2 Faculty of Mechanical Engineering and Naval Architecture, University of Zagreb, 10000 Zagreb, Croatia; ana.pilipovic@fsb.hr; 3 Faculty of Biomedical Sciences, Università della Svizzera Italiana, 6900 Lugano, Switzerland; ortho@gfilardo.com; 4 Department of Forensic Medicine and Criminology, School of Medicine, University of Zagreb, 10000 Zagreb, Croatia; slavica.martinovic@mef.hr (S.M.); anita.galic@mef.hr (A.G.M.); 5 Department of Orthopedics and Traumatology, Medical Park Gebze Hospital, 41400 Kocaeli, Turkey; basalozgur@gmail.com; 6 Orthopaedic Department, School of Medicine, University of Zagreb, 10000 Zagreb, Croatia

**Keywords:** anterior cruciate ligament reconstruction, biomechanical phenomena, hamstring tendon graft, tendon twisting

## Abstract

**Background:** Evidence regarding the effects of twisting hamstring tendons on graft properties in anterior cruciate ligament (ACL) reconstruction remains controversial. The objective of this study was to evaluate the influence of a 180° twist on the tensile properties of human hamstring tendon grafts (HTGs). **Methods:** Fourteen human cadavers were included, and hamstring tendons (semitendinosus [ST] and gracilis [GR]) were harvested bilaterally. Matched pairs of tendons were allocated to the ST and GR groups and further subdivided into control (ST-0, GR-0) and experimental (ST-180, GR-180) subgroups. Standard tripled single-tendon grafts were prepared in the control groups, while grafts in the experimental groups were twisted by 180°. All grafts were preconditioned and tested using a universal testing machine (Shimadzu AGS-X, Shimadzu Corporation, Japan) to determine tensile strength, stiffness, tensile modulus, and energy absorption capacity. **Results:** In the semitendinosus group, the maximum force was 648.72 (±287.71) N for ST-0 and 853.11 (±189.14) N for ST-180, with energy absorption capacities of 9.21 (±4.47) J and 13.48 (±4.95) J, respectively. Although the mean values of the investigated parameters were consistently higher in the ST-180 group, these differences did not reach statistical significance. In the gracilis group, no statistically significant differences were observed between the GR-0 and GR-180 subgroups for any parameter. **Conclusions:** Twisting hamstring tendons by 180° during graft preparation results in limited alterations of biomechanical properties, without statistically significant improvements. These findings call into question the clinical relevance of this technique in enhancing graft material properties for ACL reconstruction.

## 1. Introduction

Anterior cruciate ligament (ACL) tears are among the most common knee injuries, with an annual incidence of approximately 75 per 100,000 individuals [1,2]. They occur predominantly in young, physically active populations and account for nearly half of all knee injuries [3]. Over the past two decades, the incidence of ACL tears has steadily increased, largely due to the rising participation of children and young adults in recreational and competitive sports [4]. This trend is expected to continue [4,5]. If left untreated, ACL tears can result in chronic knee instability, which in turn predisposes patients to early degenerative changes in the knee joint, namely osteoarthritis (OA). It is estimated that up to 50% of individuals with an ACL tear will develop symptomatic knee OA within 10 to 20 years following injury [6]. Consequently, ACL injuries represent both a significant healthcare burden and a societal problem [7].

The standard surgical treatment for ACL tears is reconstruction of the ruptured ligament using specifically prepared grafts to restore functional knee stability [8]. ACL reconstruction is one of the most frequently performed procedures in orthopaedic surgery [9]. While clinical outcomes in terms of knee stability are generally favourable, degenerative changes often develop in the mid-term follow-up period, even after technically successful reconstruction [10]. Recent studies suggest that these degenerative changes may result from prolonged low-grade inflammation that persists for years following ACL reconstruction [11].

The native ACL has an ultimate load of approximately 2160 N and stiffness of 242 N/mm in young cadaveric specimens, and most commonly used autografts (BPTB, quadrupled hamstrings, quadriceps tendon) meet or exceed these structural properties in biomechanical testing [12,13,14]. Clinical series and systematic reviews emphasize that most graft failures are due to multifactorial causes, including tunnel malposition, high posterior tibial slope, concomitant unaddressed pathology, biological healing limitations, and premature return to sport, rather than insufficient graft strength per se [15,16,17].

Evidence where reduced graft quality correlates with higher failure risk includes small-diameter hamstring autografts (<8 mm), which have been associated with higher revision rates in some cohorts, although recent studies question a universal threshold [18,19,20]. Similarly, allografts processed with irradiation or chemical sterilization demonstrate inferior mechanical properties and increased clinical failure rates, particularly in young and active patients [21,22]. During the early ligamentization phase, grafts transiently lose structural properties, supporting the need for adequate initial margins of strength, but clinical failures in this phase remain primarily related to biology, fixation, and load exposure [23].

Therefore, while ensuring sufficient graft diameter and avoiding mechanically compromised tissue (e.g., over-processed allograft) is clinically relevant, there is no evidence that increasing graft strength beyond the levels already achieved by standard autografts independently reduces surgical failure. Instead, proper tunnel placement, secure fixation, biological integration, and risk factor modification remain the main determinants of successful ACLR outcomes. However, in light of emerging evidence regarding the influence of tendons used as autografts for ACL reconstruction on knee kinematics and, consequently, joint stability, the present study will focus exclusively on the tendon properties, in order to ensure a more precise evaluation of their biomechanical potential.

The most widely used grafts for ACL reconstruction are hamstring tendon grafts (HTGs) and bone–patellar tendon–bone (BPTB) autografts [23]. Less commonly used options include quadriceps tendon autografts, allografts, and synthetic grafts [24]. Each graft type has distinct advantages and limitations, and the optimal choice of graft remains a subject of debate [24,25,26].

HTGs are typically prepared from the semitendinosus and gracilis tendons, most often in the form of quadrupled (four-strand) grafts [27]. They have become increasingly popular among orthopaedic surgeons because of their lower complication rates, particularly with respect to anterior knee pain, when compared with BPTB grafts [24]. However, HTGs are associated with a higher risk of graft failure than BPTB grafts [25,28,29,30]. Furthermore, harvesting both the semitendinosus and gracilis tendons alters knee kinematics due to their important proprioceptive role in knee motion [24]. Since the hamstrings also act as agonists of the ACL, their absence may further increase the risk of graft rupture [31]. To minimize the adverse effects on knee kinematics associated with harvesting both tendons, some authors advocate the use of a single hamstring tendon for graft preparation [31,32,33]. Although this strategy is advantageous for preserving knee function, it reduces graft diameter and alters the tensile properties of the graft. Biomechanical studies have demonstrated a positive correlation between graft diameter and tensile strength [34]. Increasing the number of strands enlarges graft diameter and enhances tensile properties [4,27]. However, when only one hamstring tendon is available, the number of strands is limited by tendon length [35]. This creates a clinical need to optimize graft preparation techniques to improve tensile properties when using a single tendon.

The concept of twisting to enhance material properties originates from mechanical engineering, particularly in rope manufacturing. Ropes are composed of multiple strands twisted around their axis to improve load-bearing capacity. Given that ACL tendon grafts are also constructed from multiple strands, a logical question arises: could twisting improve graft biomechanical properties as well? To date, only a limited number of studies have examined the effects of twisting on human tendon grafts [36,37,38], and their findings remain contradictory. Therefore, the aim of this study was to investigate the effect of a 180° twist on the tensile properties of human HTGs. We hypothesized that twisting the tendon during graft preparation would improve its tensile properties.

## 2. Materials and Methods

### 2.1. Ethical Approval

The study was approved by the Ethical Committee of the University of Zagreb School of Medicine (Class: 641-01/21-02/01, No.: 380-59-10106-21-111/101, April 2021). All procedures were conducted in accordance with ethical standards and institutional regulations.

### 2.2. Specimen Collection and Preparation

**Figure 1:**
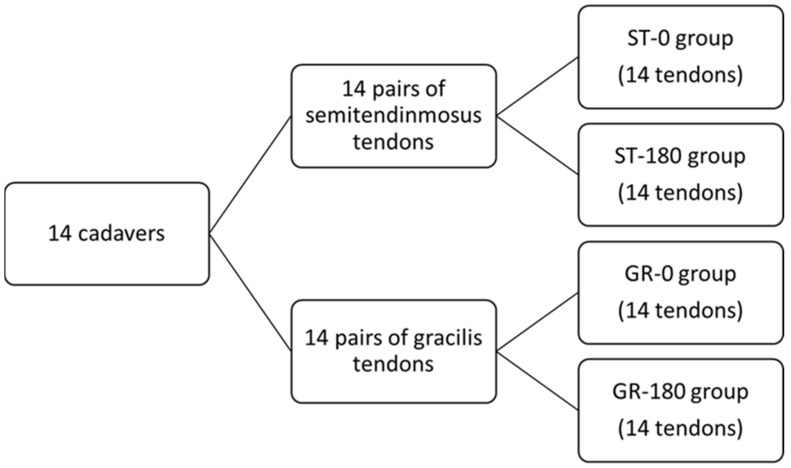
Flowchart of the study design.

This biomechanical study was conducted in two phases. In the first phase, specimens were obtained from deceased human donors at the University of Zagreb School of Medicine, Department of Forensic Medicine and Criminology, after informed consent had been obtained from the donors’ families. A total of 14 human cadavers were included. From each cadaver, semitendinosus (ST) and gracilis (GR) tendons were harvested bilaterally (Figure 1). Tendon harvesting was performed in a standardized manner through a vertical skin incision at the anteromedial crest of the proximal tibia [39]. After incision of the subcutaneous tissue, the sartorius fascia was exposed and divided by a horizontal incision. The ST and GR tendons were then identified and harvested using a tendon stripper. Following harvest, tendons were wrapped in saline-soaked gauze and stored at −20 °C until biomechanical testing [40].

### 2.3. Graft Preparation and Grouping

**Figure 2:**
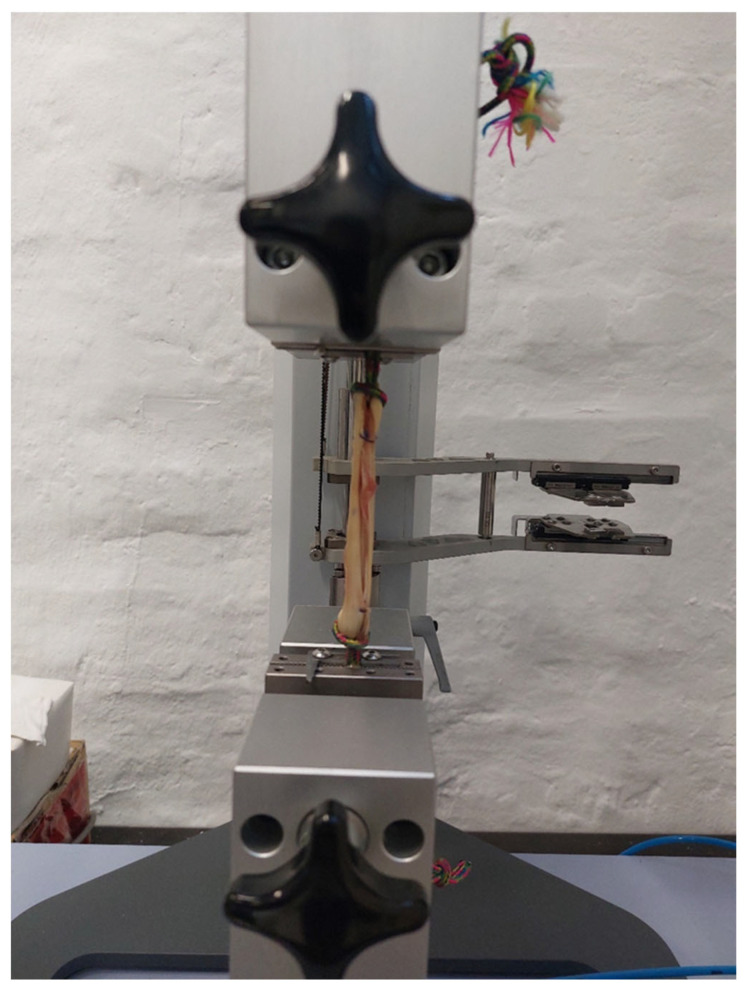
Standard tripled semitendinosus graft.

**Figure 3:**
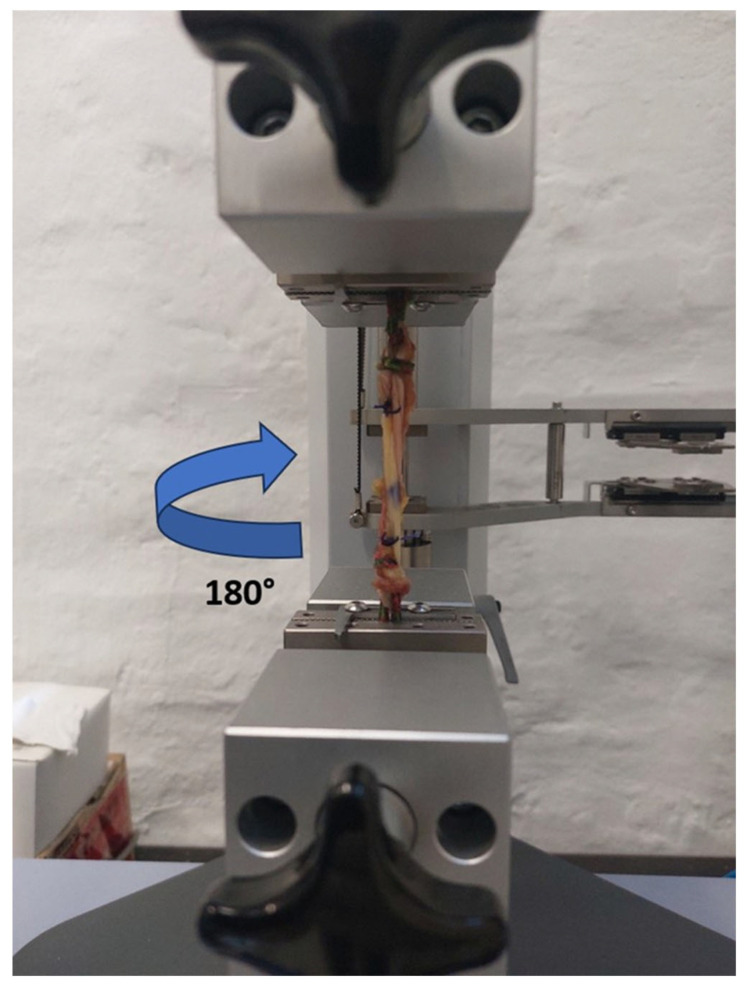
Tripled semitendinosus graft twisted by 180°.

In the second phase, biomechanical testing was performed at the University of Zagreb Faculty of Mechanical Engineering and Naval Architecture. Prior to testing, specimens were removed from storage and allowed to thaw at room temperature for at least 12 h [41]. Matched pairs of hamstring tendons were separated into ST and GR groups and further subdivided into control (ST-0 and GR-0) and experimental (ST-180 and GR-180) subgroups. In the control groups, standard tripled tendon grafts were prepared and tested, whereas in the experimental groups, tripled tendon grafts were twisted 180° along their entire length before testing. This design ensured equal distribution of specimens across all experimental conditions and enabled matched-pair analysis to evaluate the biomechanical effects of tendon twisting. In the control subgroup (ST-0), a standard tripled ST tendon graft was prepared as previously described [42] (Figure 2). In the experimental subgroup (ST-180), the tripled ST graft was twisted 180° along its entire length (Figure 3). To prevent untwisting, a surgical knot was secured at both ends of the graft. In the GR groups, the grafts were prepared using the same protocol. All tendons were mounted on the testing device using ropes secured with self-tightening knots to prevent graft slippage. Graft diameter was measured with a graft sizer, while tendon mass and length were recorded prior to graft preparation. For the purpose of measuring the initial surface area, or diameter of the tendons (straight and twisted), a special gauge was prepared (graft sizer). The gauge was made of metal milled to the exact diameters. The diameters ranged from 5.0 to 9.0 mm with a step of 0.2 mm. The diameter was recorded in the way that each tendon needed to pass through a certain hole/diameter.

### 2.4. Biomechanical Testing

Tensile testing was performed using a universal testing machine (Shimadzu AGS-X, Shimadzu Corporation, Kyoto, Japan) with a maximum load capacity of 10 kN. After preconditioning, which consisted of cyclic loading for 10 cycles up to 30 N, specimens were tested to failure at a constant speed of 5 mm/min [43,44]. All tests were conducted at room temperature (22 °C). Maximum force was recorded.

Force–displacement and stress–strain curves were generated using the integrated testing software, and the following biomechanical parameters were calculated: tensile strength, stiffness, tensile modulus, and energy absorption capacity.

***Tensile strength*** *σ_m_* [N/mm^2^] was defined as the stress sustained by the tendon at maximum force and calculated using the equation:

(1) $$\sigma_{m}=\frac{F_{max}}{A_{0}}.$$

*F_max_* [N] is the maximum force of the tendon graft and can be determined from the force–displacement diagram curve. *A*_0_ [mm^2^] represents the initial surface of the tendon and is calculated using the equation:

(2) $$A_{0}=\frac{d^{2}\cdot \pi }{4}.$$

where *d*^2^ [mm] represents the tendon graft diameter measured by graft sizer.

***Stiffness*** was defined as the resistance to deformation and obtained from the slope of the linear portion of the force–displacement or stress–strain curve. The values were measured for stresses from 2 N/mm^2^ and 4 N/mm^2^.

***Tensile modulus*** *E* [N/mm^2^] reflected the rigidity of the material and was calculated using:

(3) $$E=\frac{\sigma }{\varepsilon }=\frac{\sigma_{2}-\sigma_{1}}{\varepsilon_{2}-\varepsilon_{1}}.$$

where *σ* [N/mm^2^] represents tensile stress and *ε* [%] represents strain. Alternatively, it was derived from the slope of the stress–strain curve. The values were measured for stresses of 2 N/mm^2^ and 4 N/mm^2^.

***Energy absorption capacity (toughness)*** is the amount of energy that a material can absorb before failure and is determined by the area under the stress–strain curve. Considering the tendon behaviour, tendon energy absorption capacity is determined by the area between two points on the stress–strain curve. The starting point was the beginning of the testing and the end point was the maximum force.

### 2.5. Statistical Analysis

Statistical analysis was conducted using R Statistical Software (Version 4.2.0). The Shapiro–Wilk test was applied to assess the normality of data distribution. Tendon mass and length, as well as graft length and diameter, were compared using paired *t*-tests. Since the distribution of maximum force, tensile strength, stiffness, tensile modulus, and toughness was partially non-parametric, both the paired *t*-test and the Wilcoxon signed-rank test were applied. A *p* value < 0.05 was considered statistically significant.

## 3. Results

Out of the 14 cadavers included in this study, 11 were male and 3 were female. The mean age at the time of death was 54.2 years (range, 21–72 years). The mean storage time of the specimens prior to testing was 82 days (range, 63–102 days). No significant differences were observed in tendon mass or length, nor in graft mass or length, in either the ST or GR groups (Table 1 and Table 2).

**Figure 4:**
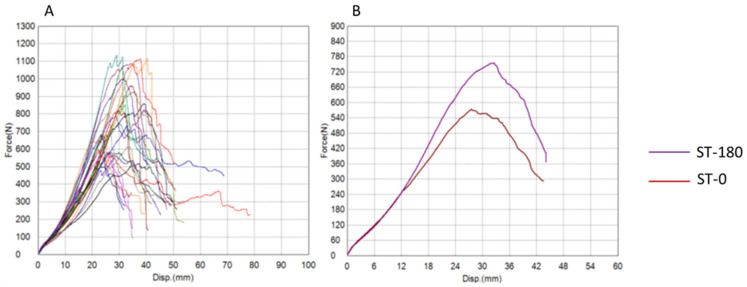
**Force–displacement diagrams for the semitendinosus group.** (**A**) Curves for all tested tendons. (**B**) Mean curves for ST-180 (purple) and ST-0 (red). The ST-180 group shows a steeper initial slope, indicating higher mean maximum force, tensile strength, and stiffness, although differences were not statistically significant (ST-180 = experimental, twisted; ST-0 = control, plain; N = newton, mm = millimeter).

**Figure 5:**
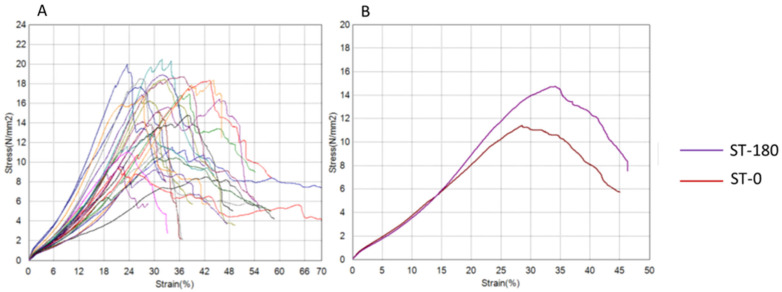
**Stress–strain diagrams for the semitendinosus group.** (**A**) Curves for all tested tendons. (**B**) Mean curves for ST-180 (purple) and ST-0 (red). The ST-180 group shows a slightly steeper initial slope, suggesting higher tensile modulus and toughness, although differences were not statistically significant (ST-180 = experimental, twisted; ST-0 = control, plain; N = newton, mm = millimeter).

**Figure 6:**
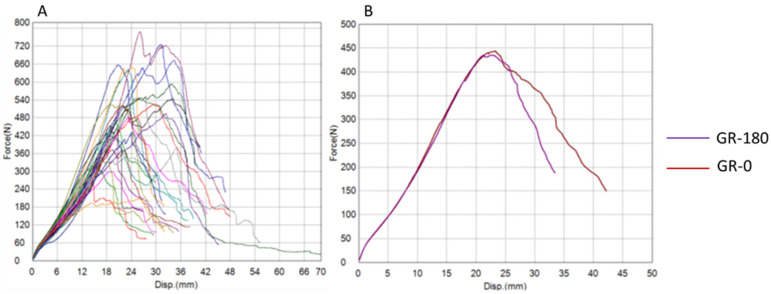
**Force–displacement diagrams for the gracilis group.** (**A**) Curves for all tested tendons. (**B**) Mean curves for GR-180 (purple) and GR-0 (red), showing similar force increase to maximum loading, indicating comparable stiffness and tensile strength (GR-180 = experimental, twisted; GR-0 = control, plain; N = newton, mm = millimetre).

**Figure 7:**
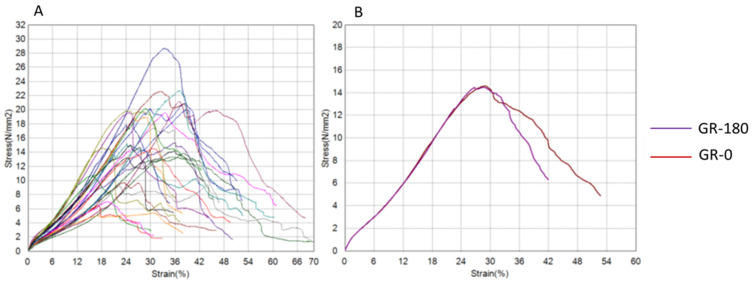
Stress–strain diagrams for the gracilis group. (**A**) Curves for all tested tendons. (**B**) Mean curves for GR-180 (purple) and GR-0 (red), showing similar initial slopes and indicating comparable elastic behaviour (GR-180 = experimental, twisted; GR-0 = control, plain; N = newton, mm = millimetre).

The results of tendon graft testing are presented graphically using force–displacement and stress–strain diagrams for both groups (Figure 4, Figure 5, Figure 6 and Figure 7) recorded during testing. The diagrams show several peaks because tendons behave like a rope, so when individual strands break, a brief drop and increase in force is recorded during tensile testing. However, the maximum force was used to calculate the most important strength in this testing (tensile strength).

**Figure 8:**
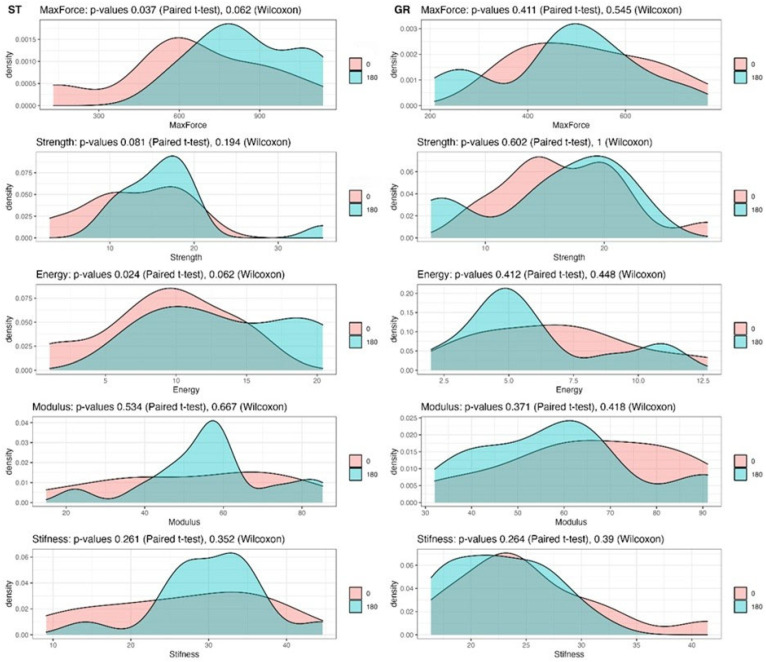
**Density plots of biomechanical parameters for semitendinosus (ST) and gracilis (GR) tendon grafts.** This figure presents the distribution of biomechanical parameters—maximum force, strength, energy absorption, modulus, and stiffness—measured in control (0) and 180° twisted (180) tendon graft groups for semitendinosus (ST) and gracilis (GR) tendons. Statistical comparisons were performed using paired *t*-tests and Wilcoxon signed-rank tests, with corresponding *p*-values provided for each parameter. Semitendinosus tendons (ST): The density plots show shifts in distributions favouring the 180° group for maximum force and energy absorption, with paired *t*-test *p*-values of 0.037 and 0.024, respectively, indicating potential improvements. However, Wilcoxon test results did not confirm statistical significance. Other parameters, such as strength, modulus, and stiffness, exhibited overlapping distributions with *p*-values > 0.05 in both statistical tests. Gracilis tendons (GR): Across all parameters, distributions for the 0 and 180° groups largely overlap, with no statistically significant differences observed (*p* > 0.05 for both paired *t*-tests and Wilcoxon tests). These results suggest that twisting the grafts may influence certain biomechanical properties, particularly for semitendinosus tendons, but with limited statistical significance. Gracilis tendons do not appear to benefit from this intervention based on the measured parameters.

The numerical values of the examined parameters are presented in Table 3 for the semitendinosus group and in Table 4 for the gracilis group. The results of the statistical analyses are also illustrated graphically (Figure 8).

The mean values of maximum force and energy absorption capacity were higher in the ST-180 group. These differences reached statistical significance when analysed using the paired *t*-test (*p* = 0.037 and *p* = 0.024, respectively). However, because the distribution of values was partially non-parametric, the Wilcoxon signed-rank test was considered more appropriate, and this analysis did not confirm statistical significance (*p* = 0.062).

For the remaining parameters—tensile strength, tensile modulus, and stiffness—the mean values were also higher in the ST-180 group. Nevertheless, these differences did not reach statistical significance (Table 3, Figure 8).

When comparing the GR-0 and GR-180 groups, no statistically significant differences were observed for any of the examined parameters (Table 4, Figure 8).

## 4. Discussion

Manipulation of hamstring tendons during graft preparation by 180° twisting affected biomechanical parameters only in the thicker tendons (ST), whereas no effect was observed in the thinner gracilis tendons (GR). The changes observed in the ST group did not reach statistical significance, making the clinical relevance of such improvements questionable for ACL reconstruction.

Since no statistically significant differences were demonstrated between the control and experimental groups, the initial hypothesis that twisting enhances graft tensile properties was not confirmed. Nevertheless, several observations warrant attention. Although not statistically significant, the mean values of all investigated parameters were consistently higher for the twisted grafts in the ST group (Table 3). Conversely, in the GR group, mean values were higher in the untwisted grafts (Table 4). Moreover, the absolute differences between twisted and untwisted grafts were greater in the ST than in the GR groups. While these findings lack statistical confirmation, they suggest that potential effects of twisting may differ between semitendinosus and gracilis tendons and merit further investigation in larger cohorts. In addition, the possibility that other twist angles, beyond 180°, could more substantially influence tendon properties should not be excluded.

To date, only two experimental [37,38] and one theoretical study [39] have investigated the effects of twisting on human tendons, and their findings are contradictory. In 2003, Ferretti et al. compared plain quadrupled hamstring grafts with grafts twisted by 720° [37]. They reported a mean maximum load of 2428.3 ± 475.4 N in the twisted group versus 1709.3 ± 581.9 N in the control group, and mean stiffness values of 310.3 ± 97.3 N/mm and 213.6 ± 97.3 N/mm, respectively—suggesting a clear biomechanical advantage of twisting. In the same year, however, an American group evaluated 180° twisting and reported the opposite: the twisted grafts had a lower mean failure load (2215 ± 775 N) compared with controls (3000 ± 563 N), and reduced stiffness (384 ± 96 N/mm vs. 675 ± 143 N/mm), concluding that twisting compromised graft properties [38].

Our results are not directly comparable with either of these studies. The maximal force recorded in our strongest group (ST-180: 853.11 ± 189.14 N) was markedly lower than those reported in both previous studies, and stiffness values were approximately ten-fold lower (ST-180: 30.30 ± 6.96 N/mm). While lower tensile properties are expected in single-strand hamstring grafts compared with quadrupled grafts, the magnitude of this discrepancy suggests that methodological factors, including differences in preparation and testing protocols, may account for the variation. Importantly, the aim of the present study was not to compare single- and double-tendon grafts but specifically to assess the effect of 180° twisting on single-tendon grafts.

In addition to these experimental studies, Sidwell et al. developed a mathematical model to simulate hamstring tendon graft behaviour during knee motion [39]. Their findings indicated that twisting HTGs by 360° did not improve tensile properties, which is consistent with the results of the present study despite the methodological differences.

The present investigation represents, to our knowledge, the first study to examine the effect of twisting on single hamstring tendon grafts. A major strength lies in the use of paired human tendons, which, although more difficult to obtain than animal tissue, provide results that are more directly translatable to clinical practice.

Several limitations should be acknowledged. Fixation of tendons during biomechanical testing is a major challenge, as slippage can compromise the results. Since there is no consensus on the optimal fixation method, we used a technique in which tendons were mounted between clamps with the aid of a rope (Figure 2 and Figure 3) to simulate double-suspensory graft fixation. Although no visible slippage occurred, the potential influence of this fixation technique on test results remains uncertain and warrants further validation. Moreover, while this study did not support the use of 180° twisting to enhance graft tensile properties, future research should investigate larger samples, different twist angles, and standardized testing protocols to better clarify whether twisting could contribute to optimization of hamstring grafts for ACL reconstruction.

## 5. Conclusions

This biomechanical study demonstrated that manipulating the hamstring tendons during graft preparation by 180° twisting affects biomechanical tissue parameters only limitedly, without reaching statistically significant differences. These findings suggest that 180° twisting does not provide a clinically relevant advantage in ACL reconstruction. Further studies with larger sample sizes and different twist angles are warranted to better define the potential role of tendon twisting in graft optimization.

## Figures and Tables

**Table 1:** Morphometric characteristics of semitendinosus tendons and their corresponding grafts in the ST-0 and ST-180 groups.

Semitendinosus Group	ST-0	ST-180	*p*-Value
Tendon mass (g)	5.03 ± 1.6	5.06 ± 1.9	0.965
Tendon length (cm)	30.113 ± 3.261	29.831 ± 2.814	0.288
Graft length (cm)	6.711 ± 1.23	6.558 ± 1.121	0.479
Graft diameter (mm)	8.15 ± 0.82	8.11 ± 0.88	0.9251

No statistically significant differences were observed between the groups across the measured parameters. Tendon mass, tendon length, graft length, and graft diameter were comparable, with *p*-values consistently above the threshold for significance (*p* > 0.05). These findings indicate that a 180° twist had no measurable effect on the morphometric properties of the grafts. (g = gram, cm = centimeter, mm = millimeter).

**Table 2:** Morphometric characteristics of gracilis tendons and their corresponding grafts in the GR-0 and GR-180 groups.

Gracilis Group	GR-0	GR 180-0	*p*-Value
Tendon mass (g)	2.345 ± 0.92	2.44 ± 0.9	0.8
Tendon length (cm)	25.808 ± 2.612	25.663 ± 2.438	0.611
Graft length (cm)	5.566 ± 1.003	5.355 ± 0.787	0.5031
Graft diameter (mm)	6.240 ± 0.793	6.340 ± 0.828	0.4854

No statistically significant differences were found between the groups in tendon mass, tendon length, graft length, or graft diameter (all *p*-values > 0.05). Both groups exhibited highly comparable values, suggesting that 180° twisting did not influence the fundamental morphometric characteristics. (g = gram, cm = centimeter, mm = millimeter).

**Table 3:** Mean values ± SD of the examined tensile properties of tendon grafts in the ST-0 and ST-180 groups.

Semitendinosus Group (ST)	ST-0	ST-180	*t*-Test (*p*)	Wilcoxon (*p*)
**Maximum Force (N)**	648.72 ± 287.71	853.11 ± 189.14	0.037	0.062
**Tensile Strength (N/mm^2^)**	12.74 ± 5.71	16.87 ± 6.23	0.081	0.194
**Energy (J)**	9.21 ± 4.47	13.48 ± 4.95	0.024	0.062
**Tensile Modulus (N/mm^2^)**	52.11 ± 21.84	56.68 ± 16.02	0.534	0.067
**Stiffness (N/mm)**	26.57 ± 10.23	30.30 ± 6.96	0.261	0.352

Maximum force was significantly higher in the ST-180 group as determined by the *t*-test (*p* = 0.037), with the Wilcoxon test showing a trend toward significance (*p* = 0.062). Energy absorption was also significantly greater in the ST-180 group (*t*-test *p* = 0.024). Although the tensile strength, elastic modulus, and stiffness values were higher in ST-180, these differences did not reach statistical significance (*p* > 0.05). These findings suggest that 180° graft twisting may positively influence force transmission and energy absorption capacity in semitendinosus grafts. (N = newton, N/mm^2^ = newton/millimetre, J = joule).

**Table 4:** Mean values ± SD of the examined tensile properties of tendon grafts in the GR-0 and GR-180 groups.

Gracilis Group (GR)	GR-0	GR-180	*t*-Test (*p*)	Wilcoxon (*p*)
**Maximum Force (N)**	506.81 ± 139.52	474.36 ± 148.14	0.411	0.545
**Tensile Strength (N/mm^2^)**	16.44 ± 5.33	15.71 ± 6.02	0.602	1
**Energy (J)**	6.52 ± 3.02	5.88 ± 2.77	0.412	0.448
**Tensile Modulus (N/mm^2^)**	66.25 ± 17.75	58.97 ± 17.58	0.371	0.418
**Stiffness (N/mm)**	25.17 ± 6.5	22.66 ± 4.45	0.264	0.39

None of the evaluated parameters showed statistically significant differences between groups, based on both *t*-tests and Wilcoxon tests (*p* > 0.05 for all variables). Maximum force, tensile strength, energy absorption, tensile modulus, and stiffness were all comparable. These findings indicate that 180° twisting did not significantly affect the mechanical performance in the gracilis group. (N = newton, N/mm^2^ = newton/millimetre, J = joule).

## Data Availability

The original contributions presented in this study are included in the article. Further inquiries can be directed to the corresponding author.
